# Mindfulness and Wellbeing Among College Students During the COVID-19 Pandemic: A Qualitative Analysis of Emergent Themes and Concerns

**DOI:** 10.7759/cureus.20755

**Published:** 2021-12-27

**Authors:** Julianna A Rava, Emily Hotez

**Affiliations:** 1 Public Health/Epidemiology, Fielding School of Public Health, University of California Los Angeles, Los Angeles, USA; 2 Internal Medicine, University of California Los Angeles David Geffen School of Medicine, Los Angeles, USA

**Keywords:** med-peds, college, health, mindfulness, emerging adulthood

## Abstract

Emerging adulthood (ages of 18-30 years) is a critical developmental period characterized by mental health challenges, particularly for college students who experience distinct mental health issues. Mindfulness-based approaches have been associated with mental health benefits. This study aimed to assess the mental health and wellbeing of college students with qualitative data obtained via their participation in a mindfulness exercise. We analyzed the sentiments and concerns of college students nearly a year into the coronavirus disease 2019 (COVID-19) pandemic. The results led to the development of four major themes: a source code of the COVID-19 pandemic, assessments of mindfulness and wellbeing, emergent concerns, and coping strategies. The findings from this paper can inform combined internal medicine and pediatrics (Med-Peds) providers’ efforts to improve the mental and physical health outcomes among emerging adults.

## Introduction

Emerging adulthood (ages of 18-30 years) represents a critical developmental period associated with mental health challenges, especially among college students who experience distinct mental health issues. In general, the US undergraduate population has seen considerable increases in mental health diagnoses and utilization of mental health services over the past decade. Recent data shows that over a third of college students have had a mental health diagnosis; specifically, there have been notable increases in anxiety, panic attacks, depression, and suicidal ideation among undergraduate students [1,2]. Despite these increases in mental health diagnoses, college students are underutilizing services and interventions, which are compounding their mental health challenges [1]. Many college students are emerging adults, and this period coincides with the transition from pediatric to adult care [3]. As a result, combined internal medicine and pediatrics (Med-Peds) providers must be attuned to these mental health challenges.

The ongoing coronavirus disease 2019 (COVID-19) pandemic has led to global turmoil, exacerbated by a polarized US Presidential election, and racial and social justice unrest. During this time, undergraduate students have attempted to maintain their academic obligations while they have been impacted by these external stressors. Not surprisingly, they have reported higher rates of depressive and anxiety-related symptoms over the course of the pandemic [4-7]. Recent studies have found that between 57-90% of students reported that they were unable to cope adequately with the stress related to the current situation [4,7]. Also, students have reported that they were experiencing negative physical health outcomes due to COVID-19-related worries that increased anxiety and depressive thoughts [6]. Med-Peds providers, therefore, face further challenges to support their emerging adult patients.

In recent years, there have been increased efforts to integrate mindfulness-based strategies into Med-Peds clinics [8]. Mindfulness can take on different meanings but is often defined as living in the present moment, on purpose and nonjudgmentally [9]. There are multiple approaches to practicing mindfulness, and the most common approaches are a body scan, meditation, mindful seeing, conscious listening, cognizant breathing, and the five senses exercise (notice five things you can see, four things you can feel, three things you can hear, two things you can smell, one thing you can taste). A mindfulness session may focus on one of these strategies or incorporate more than one. Additionally, there are standardized mindfulness-based interventions: mindfulness-based stress reduction (MBSR) and mindfulness-based cognitive therapy (MBCT). There are significant mental health benefits associated with practicing mindfulness, such as reducing anxiety and stress and decreasing mood-related symptoms [10-12]. Research has found that college students may find significant benefits in practicing mindfulness. Indeed, many students who use mindfulness in their daily lives or as an intervention have reported reduced anxiety and depression symptoms and healthier habits that improve overall physical health [13-16]. Unfortunately, there has been little effort to incorporate mindfulness practices into Med-Peds treatment strategies. Given the pronounced mental health challenges that have emerged during the pandemic for this population, mindfulness may be more important than ever.

The current study aims to describe the mental health and wellbeing of college students based on their participation in a mindfulness exercise. Using qualitative analysis, we present emergent themes and concerns among college students nearly a year into the COVID-19 pandemic. The findings from this paper can inform Med-Peds providers about the benefits of mindfulness-based approaches that could potentially improve mental and physical health outcomes among college students.

## Materials and methods

Sample and study design

This study included students enrolled in an undergraduate-level course at a four-year public university in Southern California. The course was approved as a research study by the academic institution’s Institutional Review Board (IRB# 20-000320). The course took place during the Winter 2021 academic quarter and lasted 10 weeks. The data used for this study were considered part of a required assignment for the course and expected to be completed by all enrolled students; one student did not complete the qualitative assignment (98% response rate). All data were collected in January 2021.

The sample’s demographic characteristics are presented in Table 1. The sample (n=55) was representative of the host institution with respect to race (Asian/Native Hawaiian/Pacific Islander=42%, White=22%, Multi-racial or other race=20%, Black/African American=5%), and ethnicity (Latinx=22%). Over a fifth of students self-reported a disability (22%). The majority of the sample were in their senior years of study - third or fourth academic years (42% and 53%, respectively), and female (62%). At the time of the survey, 76% of students identified as dependents of parents/caregivers. Students’ household income varied, with 20% reporting less than $25,000 annually and 38% reporting $100,000 or more annually.

**Table 1:** Demographic characteristics of college students ADD/ADHD: attention deficit disorder/attention deficit hyperactivity disorder; OCD: obsessive-compulsive disorder

Characteristics	Values, n (%)
Year in undergrad	
2nd year	3 (5%)
3rd year/transfer	23 (42%)
4th year/transfer	29 (53%)
Gender identity	
Man/trans man	18 (33%)
Woman/trans woman	34 (62%)
No response	3 (5%)
Race	
White American	12 (22%)
Black or African American	3 (5%)
Asian American, Native Hawaiian, or Other Pacific Islander	23 (42%)
Other	11 (20%)
No response	6 (11%)
Hispanic ethnicity	12 (22%)
Household income	
Less than $25,000	11 (20%)
$25,000-60,000	9 (16%)
$60,000-100,000	9 (16%)
$100,000-250,000	16 (29%)
More than $250,000	5 (9%)
No response	5 (9%)
Dependent	42 (76%)
Self-reported disability	
No disability	43 (78%)
Chronic illness (cancer, diabetes, autoimmune disease, etc.)	3 (6%)
Mental health (depression, anxiety, OCD, etc.)	4 (7%)
Physical health (speech, sight, hearing, mobility, etc.)	1 (2%)
Learning (dyslexia, ADD/ADHD, etc)	4 (7%)

Qualitative data and analysis

For the qualitative data collection, students were assigned to write a journal entry about practicing mindfulness in an outdoor space. The assignment prompt was as follows: Bring a blank piece of paper or a blank notebook out to a green space in your neighborhood. Notice what you feel, hear, and experience. Write down whatever thoughts that come to mind and what you are experiencing. When you return indoors, take a photo of your journal entry and upload it.

The study’s authors performed thematic analysis on the journal entries [17]. The first author developed a preliminary coding scheme using line-by-line coding within the responses [18]. Line-by-line coding aligns a code for each line of the qualitative data. The final coding scheme was decided between two coders (the study’s authors), which resulted in 10 major codes and two minor codes (Table 2). One of the coders was a doctoral-level researcher with experience in qualitative analysis, and the other was a faculty member with a background in qualitative methods and emerging adulthood. Two coders were used for 20% of the responses, and interrater reliability had an average kappa of 91.4%; the lead author coded the remaining excerpts for thematic analysis. Coding was executed using the DeDoose software.

## Results

**Figure 1:**
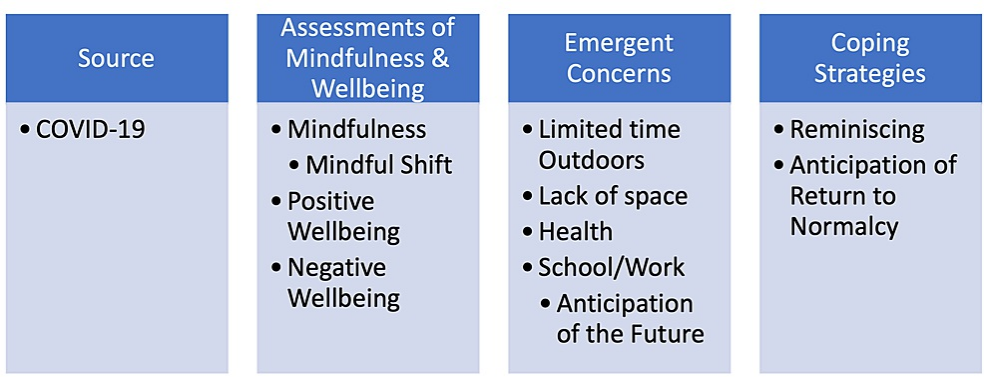
Thematic coding structure

Thematic coding of the mindfulness journal activity led to the 12 codes being categorized into four major themes: a source code of the COVID-19 pandemic, assessments of mindfulness and wellbeing (mindfulness, mindful shift, positive wellbeing, negative wellbeing), emergent concerns (limited time outdoors, lack of space, health-related, school- or work-related, anticipation of the future), and coping strategies (anticipation of a return to normalcy, and reminiscing) (Figure 1). Code definitions are presented in Table 2. Students’ journal entries were frequently coded for more than one theme. For example, students’ entries generally had a code for assessments of mindfulness and wellbeing and emerging concerns. Additionally, some students may have had multiple codes within the same theme; if they expressed concerns related to lack of space and health concerns, each code was recorded.

**Table 2:** Thematic codes and definitions Themes and codes are not mutually exclusive *Subthemes: Mindful shift is a minor code of Mindfulness and Anticipation of the Future is a minor code of School/Work

Code	Definition
Theme 1: COVID-19 pandemic	
COVID-19	Direct mention of the COVID-19 pandemic
Theme 2: assessments of mindfulness and wellbeing	
Mindfulness	Any indication that they are present in the moment; presents mindfulness to prompt by describing what they see, hear, or feel in an outdoor space; expresses any feelings directly related to the moment (feelings of being peaceful, calm, relaxed, relief, happy, grateful, blessed)
Mindful shift in feelings*	Directly related to moment and recognizing a shift from negative feelings to positive due to the mindfulness exercise
Positive aspects of wellbeing	Feelings of being peaceful, calm, relaxed, relief, happy, grateful, blessed related to the present moment or while reflecting on other experiences; expressing feelings and/or emotions that demonstrate positive mental health and wellbeing
Negative aspects of wellbeing	Feelings of worry, stress, being busy, tired; expresses negative emotions or mentions poor mental health, anxiety; clarifying point: does not include mentions of negative feelings shifting to positive due to exercise
Theme 3: emergent concerns	
Limited time outdoors	Lack of being outdoors or spending too much time looking at a screen
Lack of space	Family or roommate-related inconveniences/lack of physical space; discussion of quarantining and living arrangements
School/work	Feelings of missing out on college experience; expresses feelings about school/work
Anticipation of the future*	Questions, worries, or thoughts about things in the future, often related to work or school
Health	Fear/anxiety about getting sick with COVID; fear/anxiety about family members getting sick; any mention of negative physical health for themselves or family members; any mention of themselves or family members experiencing COVID-19 symptoms
Theme 4: coping strategies	
Anticipation of return to normalcy	Reflecting on lack of normalcy; wishing for a return to normalcy; explicitly referencing a return to normal things/activities prior to pandemic (relatively recently, just prior to pandemic)
Reminiscing	Shares stories from high school, childhood - nostalgic; typically leads to sharing positive feelings/emotions; feelings of missing a person/pet/place

COVID-19 pandemic

Students often cited the COVID-19 pandemic in their journal entries. The COVID-19 code was seen as a source code and was often mentioned in relation to other codes. Students expressed feelings of isolation, loneliness, and fear due to the circumstances of the pandemic. One student noted, “I heard an ambulance, which is pretty normal since I do live next to Ronald Reagan Hospital. However, I couldn’t help to think if the increase of ambulances I have heard are due to COVID, and people needing emergency help. We have been in this pandemic for quite some time; however, it is getting so bad and in [host city], I fear for my family's life and health.”

The pandemic also had many students reflecting on the past year and expressing sentiments of gratitude. As one student stated, “After months of being holed up inside my house, being reminded of the little things of life really made me reminisce and cherish my memories prior to the pandemic.”

Assessment of mindfulness and wellbeing

Positive Wellbeing

Many students shared expressions of positive wellbeing, such as being happy, relaxed, or grateful. Most often, students conveyed statements of positive wellbeing when discussing the mindfulness exercise or when reminiscing on happier times. Examples of positive wellbeing include the following statements:

“Especially during winter, I notice that even spending just a few minutes outside is great for my mental health. Usually, it helps me feel happier, more awake, and more motivated to work on my schoolwork when I get back inside.”

“I am happy with life, I am proud of what I’ve done”

Negative Wellbeing

Expressions of negative wellbeing were considered statements of sadness, fear, poor mental health, among others. Similar to codes of positive wellbeing, students who expressed sentiments of negative wellbeing often correlated with codes from other themes. Those who expressed negative wellbeing were more likely to relate it to the pandemic or emergent concerns. Regarding the pandemic, one student noted, “It’s strange because even though there are plenty of people around me and quarantining with me, I still feel extremely lonely. I feel like COVID has taken over every conversation along with intense politics and just emotional burnout. It feels foreign now to ask a friend to 'hang out' or FaceTime to have some deep talks. It’s just a very isolating time.”

Another student emphasized the impact of external stressors on their academic pursuits and overall wellbeing: “There’s so much going on both in my personal life and in the world around me. I have to sign up for my MCAT reschedule tomorrow. My friend’s dad passed away from COVID a couple of days ago. The US capitol was attacked by rioters. There’s so much noise. Even as I sit outside, there’s all this movement and noise.”

Mindfulness

The majority of the students demonstrated mindfulness in their journal entries, although many diverted their thoughts during the journal activity at some point. Mindfulness was demonstrated as centering their thoughts on what they could hear, see, and feel at that moment of journaling. An example of mindfulness in one student's journal was as follows: “The first word that comes to mind is silence. My green space is an area of trees and flowers and plants in the middle of all the houses. Seeing the green and nature makes me feel at peace.”

Another account of mindfulness, one that does not attach any elements of wellbeing, was as follows: “It’s a perfect day to sit outside. The sun is out, but it’s not too hot and there’s a slight breeze that I notice every couple of minutes. Directly around me, I notice several other people sitting on the other benches. Most of them seem to be doing schoolwork on their laptops and a few are just sitting quietly. Directly ahead of me, I can see a group of people dancing. There are cameras, and they seem to be choreographing for a video. Other than that, all I notice is the sound of a leaf blower in the distance.”

While many students attached positive or negative expressions of wellbeing to their mindfulness exercises, some students stayed completely detached in describing their observations.

Mindful Shift

A mindful shift is a subcode of mindfulness; students must have presented mindfulness during the exercise to be eligible for a mindful shift code. Many students that presented mindfulness also expressed a mindful shift, such that, performing the mindfulness activity created a shift from negative sentiments to a positive state of wellbeing. As one student noted, “Coming outside to sit in peace and silence in such beautiful scenery was extremely relaxing and cathartic for my emotions and mind.” While another mentioned, “Here I have a chance to slow down my breathing, release the tension in my jaw, and realize that I actually feel okay. This doesn’t automatically solve every one of my problems, but stepping out into this space does feel like a small mental reset or pause that I think I could benefit from more often.”

Emergent concerns

Many students used the time during the mindfulness exercise to reflect on concerns they encounter in their daily lives. The main areas of concern highlighted by students were limited time outdoors, lack of space, health, and school/work. These codes were heavily associated with the source COVID-19 pandemic code, as these were areas of their lives impacted by the pandemic.

Limited Time Outdoors

Students noted they were spending less time outdoors due to the COVID-19 pandemic, often citing the remote learning experience. An example expressed by one student was as follows: “I haven’t been outside in about five days. Most of my time has been spent with blue light and stale air. For once I can hear something other than the squeaking of my chair and whirring of my computer.” Additionally, many students welcomed this mindfulness activity as an opportunity to spend time outside, as one student noted, “The environment feels more calming and feeling the cool air is refreshing with so much time being spent on Zoom meetings this past year, I find myself spending all my time in the same places. Either I sit at my desk or somewhere in the living room. Maybe a change of scenery for class or studying could be refreshing.”

Lack of Space

Similarly, concerns about a lack of space were usually expressed due to quarantine and moving back into a parent’s home due to university housing shutdowns. A lack of space was often associated with feelings of negative wellbeing - as students discussed crowded roommate/family situations, risks of COVID-19, and a lack of quiet needed for academic success and overall positive wellbeing. One student shared, “I am on my couch because I am staying out of my room given the COVID situation [roommate has COVID], and sleeping on the couch for the past week has taken a toll on my body - my shoulders feel heavy/tight and I feel the need to crack my back. There is a baby crying in the apartment building over and I can hear them.”

While another student stated that the mindfulness activity helped relieve some of the stress due to lack of space, “I have been home during quarantine, I do not have time alone as I am sharing spaces with my family. I feel that this time alone is giving me the opportunity to take a deep breath mentally and prepare me for my busy day ahead.” This sentiment was shared by several students who shared concerns about a lack of space.

Health

Some students mentioned either gratitude or fear for their health, family, or friends’ health; this code heavily coincided with the COVID-19 pandemic code. One student described the fear she felt as an essential worker, “I work at Ralphs, a grocery store, and ever since November I have been terrified to get sick.” While another student reflected on the pandemic and expressed gratefulness for her family’s health, “This year has been a long journey in which I am grateful to have my health and my family.”

School/Work

Many students used the journaling activity as an opportunity to reflect on the stresses of school or work. Additionally, many students expressed frustration with the remote learning experience and adjustments to the student-life environment due to the pandemic. As one student noted, “I’m grateful to still be living off-campus, but it’s just unfortunate that the student experience was ruined for everyone.” Similarly, another student shared, “I do not enjoy remote learning as it is nowhere near close to being on campus at UCLA. Being a transfer student that is set to graduate in 2021 of Spring, I was only able to experience a quarter and a half on campus, so it is a bit depressing. To add to that, I believe that our graduation will also be held online, which I was hoping we could avoid.”

Anticipation of the Future

Additionally, students who discussed school or work often noted concerns about their future and ensuring that they were meeting expectations regarding their career aspirations. This code was considered a subcode of the School/Work code and was deemed eligible for coding if that previous code was used. Students who had thoughts about the future often demonstrated a negative sense of wellbeing and cited feelings of stress or anxiousness due to the unpredictability of their current situation. One student stated: “This week I’ve been feeling very anxious. Thinking about what I want to do with my life after I graduate in March has been giving me stress because there are so many different paths and opportunities I’m not really sure what I want for myself, let alone how to get there. I’m really afraid of letting down not only myself but also my friends and family who have supported me throughout college. I hope I make the right decision.”

Coping strategies

Many students used the mindfulness activity to escape the current environment - anticipating a return to normalcy or reminiscing on happier times. These codes demonstrated a coping strategy by students; as most students stated negative feelings towards the current situation and expressed positive emotions regarding moments in the past or hopes for the future.

Anticipation of Return to Normalcy

Some students expressed wishes for a return to “normalcy”, or to a societal norm, prior to the pandemic. Others recognized a slight sense of normalcy being outdoors among strangers for the first time since the pandemic started. As one student noted, “And sitting outside has really made me look forward to the future when we are COVID-19-free and can freely roam as we did before this nightmare.”

Reminiscing

Additionally, many students used the activity to reminisce on memories of their childhood or pleasant times with loved ones that had passed. Reflecting on these memories invoked feelings of gratitude for the happier times they’ve had or enabled them to savor the positive feelings of those memories. For example, one student shared, “While looking at these parts of the park, I remember some of my memories in this park. When I was younger, my dad and I would play on the basketball courts. I also remember my high school’s baseball team playing on the baseball field. I also remember passing by these parts of the park in my cross-country practices in high school. When thinking of these experiences and looking at these parts of the park, I feel really happy. I really enjoy recollecting my childhood experiences. It's something I realize I don’t do often, and I would like to take more moments like these to notice my surroundings and connect it to past experiences of mine.”

## Discussion

This qualitative study among undergraduate students attending a large, diverse, public university aimed to identify factors impacting students’ overall wellbeing during the ongoing COVID-19 pandemic. As a course requirement, students participated in a mindfulness exercise wherein they completed journal responses to an open-ended mindfulness prompt. The exercise allowed students to share unexpected thoughts, feelings, and concerns related to the COVID-19 pandemic as well as served as an instrument to relieve and address the emotions they were experiencing.

We found several emergent themes impacting students’ overall wellbeing. Themes were often co-occurring; those who expressed negative wellbeing used the journaling activity to express concerns related to the pandemic, discontent with the remote learning experience, limited time spent outdoors since the pandemic started, and a lack of space due to the pandemic conditions. Additionally, many students used mindful journaling as a coping tool to express desires about day-to-day activities returning to normal, to reminisce on happier times, or to express gratitude or hopefulness for the future. Further, and significantly for medical providers, many students expressed a mindful shift in wellbeing while performing the mindfulness activity. Students often stated that they were stressed or in a negative space when they began the mindfulness activity but after engaging in the activity, they were less anxious, relaxed, and in a more positive mindset. Based on these findings, Med-Peds providers may recommend mindfulness-based exercises to their emerging adult patients to help them cope with the mental health challenges in the aftermath of the COVID-19 pandemic.

There have been minimal undertakings to integrate mindfulness-based strategies into mainstream clinical practice; however, several meta-analyses support the benefits of using mindfulness strategies to address mental health challenges [19-21]. Therefore, it is necessary to bridge the research to practice pipeline and implement mindfulness-based approaches in clinical practice. Such efforts would align with Med-Peds providers’ ongoing efforts to implement flexible, accessible, and timely mental healthcare for this population and to promote a multidimensional approach to mental and physical health [4].

There are several strengths to our study. The use of a qualitative approach enabled us to understand the complexity of stressors impacting undergraduate students nearly a year into the COVID-19 pandemic. Additionally, students often expressed the state of their wellbeing in relation to certain stressors or relievers associated with the pandemic; this allowed us to understand on a fundamental level what is impacting students during the pandemic and what tools are they using to cope with external stressors. Lastly, the mindfulness exercise provided an opportunity for students to express their feelings about the COVID-19 pandemic unprompted; therefore, emergent themes were spontaneous and not driven by a prepared survey.

There are some limitations to the current study. Although our sample was diverse and representative of the student body at the host institution, findings reflect the perspectives of only one university population at a single time point. Therefore, these findings should not be used exclusively to represent the undergraduate population during the COVID-19 pandemic. Rather, it will be important to aggregate the information gathered in this study with other studies’ findings during this time. Also, this study only used qualitative methods; future studies should use a mixed-methods approach and should involve subjects from more than one university. Lastly, the sample and university population, in general, had a smaller proportion of Black/African American students compared to the average US university; hence, future studies should try to include a larger sample of Black/African American student experiences during the COVID-19 pandemic.

## Conclusions

Our study demonstrated that there were COVID-19-related stressors impacting the overall wellbeing of undergraduate students in our sample. Many students used mindfulness exercise as a tool to cope with the various external stressors. Also, the majority of students expressed a mindful shift from sentiments of negative wellbeing to positive wellbeing thanks to the mindfulness activity. Therefore, Med-Peds practitioners should consider the benefits of mindfulness strategies when working with this population, particularly during times of extreme external stress on their overall wellbeing.
